# Fabrication of a novel core–shell–shell temperature-sensitive magnetic composite with excellent performance for papain adsorption†

**DOI:** 10.1039/d1ra04128b

**Published:** 2021-07-16

**Authors:** Pengfei Chen, Shun Yao, Dongmei Zheng, Zhiyuan Xu, Jinling Yu, Tingting Liang

**Affiliations:** School of Food and Bioengineering, Xihua University Chengdu 610039 People's Republic of China pengfeimj1028@163.com; School of Chemical Engineering, Sichuan University Chengdu 610065 People's Republic of China cusack@scu.edu.cn

## Abstract

Herein, a novel temperature-sensitive magnetic composite (Fe_3_O_4_@SiO_2_@P(NIPAM-*co*-VI)/Cu^2+^) with a uniform core–shell–shell structure was successfully prepared *via* a layer-by-layer method. The resulting magnetic composite revealed good magnetic properties and remarkable affinity to papain with a maximum adsorption capacity of 199.17 mg g^−1^. The adsorption equilibrium data fitted the pseudo-second-order kinetic and Freundlich models well, and the major thermodynamics parameters indicated that adsorption was an endothermic and spontaneous process. Fe_3_O_4_@SiO_2_@P(NIPAM-*co*-VI)/Cu^2+^ could thermally protect papain, which is attributed to the reversible hydrophilic–hydrophobic transition of the composite at temperatures below and above the lower critical solution temperature. More importantly, the magnetic composite could be recycled at least six times without a remarkable loss in its adsorption capacity, and the process of adsorption and elution had no significant effect on the activity and structure of papain. This work could provide a novel separation method for papain without loss of its activity.

## Abstract 2

A novel core–shell–shell temperature-sensitive magnetic composite was designed. The composites showed excellent performance for papain adsorption and could thermally protect papain.

## Graphical abstract

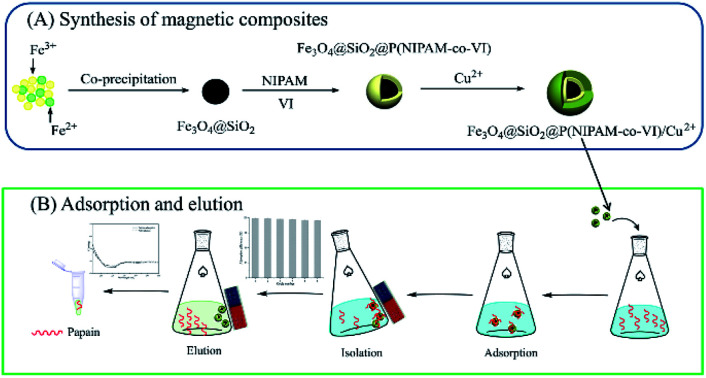

## Introduction

Demands for sustainable development and green production have resulted in the increased use of enzymes for catalytic engineering in fields such as textile treatment, food processing, and pharmaceutical ingredient synthesis.^1,2^ However, achieving the theoretical results of most enzymes in practical applications is challenging because of the lack of cost-effective schemes for the recovery and purification of soluble enzymes with high activity retention.^3,4^

Immobilized metal ion affinity chromatography (IMAC) is considered a highly effective approach for the separation and enrichment of enzymes;^5,6^ this technology relies on the interaction between metal ions and electron donor groups, such as histidine, located on the surface of enzymes. Unfortunately, conventional IMAC-based affinity chromatographic methods are time consuming and difficult to scale up; moreover, they often require sample pre-treatment and have low reaction yields.^7^

As an emerging approach in purification technology, magnetic-based separation methods, such as magnetic solid-phase extraction,^8,9^ are characterized with many attractive properties, including time savings, simplicity, easy scale-up, cost effectiveness, and good biocompatibility,^10–12^ more importantly, the technique may potentially overcome the issues described above. As an important magnetic adsorbent, core–shell-structured magnetic polymeric microspheres with Fe_3_O_4_ MNPs as the magnetic core and functional polymers as the shell demonstrate not only the enhanced stability of the magnetic particles but also favorable functional groups capable of interacting with target molecules. Thus, these microspheres are often applied to polymeric coating. A number of alternative adsorbents combining IMAC technology and magnetic polymeric microspheres have been used to separate and purify His-tagged proteins.^13–15^ Nitrilotriacetic acid (NTA) and iminodiacetic acid (IDA) are the affinity ligands most commonly used to immobilize metal ions and isolate proteins. However, these ligands are expensive and difficult to obtain. In addition, the bonded metal ions may easily be lost during sample loading and washing because they interact weakly with the ligand (*i.e.*, each metal ion can only coordinate with one IDA or NTA ligand).^5,7^ The specific enrichment of proteins by magnetic-based IMAC remains a challenging endeavor on account of the lack of conventional materials with high specificity and binding capacity.^7^ Therefore, discovering a robust ligand that can generate a continuous shell and achieve a high loading density of metal ions on the magnetic microspheres is of great significance; such a discovery could lead to higher loading capacities, better selectivity, easier separation, and lower cost for enzyme separation.

Magnetic polymeric microspheres based on poly(*N*-isopropyl acrylamide) (PNIPAM) have gained increased interest as “smart” adsorbents for the separation of proteins on account of their low critical solution temperature (LCST) in aqueous solution.^16–18^ PNIPAM has an LCST of approximately 32 °C.^19,20^ When the temperature is below the LCST, the polymer is swollen and hydrated; however, when the temperature is above the LCST, the polymer collapses and becomes dehydrated because the hydrophilic/hydrophobic balance in the polymer network structure is disrupted.^11^ However, as for used in IMAC, PNIPAM has no reactive groups that could directly be used for coupling of an affinity ligand. Reactive groups are usually introduced either by copolymerization of NIPAM with monomers containing reactive groups or the use of chain transfer agents containing reactive groups.^21^ The monomer 1-vinylimidazole (VI) has been reported to exhibit metal-uptake properties;^21,22^ and researchers believe that copolymerizing this monomer with NIPAM could produce the necessary reactive groups for IMAC. Hence, in the present work, a NIPAM copolymer with VI was first synthesized as a macro-ligand to immobilize Cu^2+^ and then used to generate a continuous shell to obtain magnetic composites.

The layer-by-layer process is a simple, versatile and elegant technique to fabricate magnetic nanoparticles with a core–shell structure.^23,24^ The main advantage of this process is that it allows the independent optimize and fine-tuning of different properties of individual components of magnetic polymeric microspheres. Thus, the properties different materials may be well combined.^24,25^ Herein, a novel magnetic composite featuring immobilized Cu^2+^ and a uniform core–shell–shell structure, namely, Fe_3_O_4_@SiO_2_@P(NIPAM-*co*-VI)/Cu^2+^, was fabricated *via* the layer-by-layer technique. The structure and property of the magnetic composites were characterized by scanning electron microscopy coupled with energy dispersive X-ray detector (SEM-EDS), transmission electron microscopy (TEM), powder X-ray diffractometer (PXRD), Fourier transform infrared spectroscopy (FT-IR), thermogravimetric analysis (TGA), differential scanning calorimeter (DSC) and a superconducting quantum interference device (SQUID).

Papain (EC 3.4.22.2) exhibits broad proteolytic activity and is widely used in food, medical, cell isolation, detergent, leather, cosmetics and pharmaceuticals.^26,27^ Which has the natural surface histidine residues and is selected as the model enzyme to evaluate the performance of the magnetic composites synthesized in this work. The optimal conditions for papain adsorption, including temperature, pH, initial concentration of the enzyme, and ionic strength, were investigated, and the adsorption performance of the magnetic composites was evaluated *via* kinetic and isothermal adsorption, recyclability, and thermal protection studies. The natural properties of papain after adsorption and elution were also investigated *via* the Folin–Ciocalteu method and circular dichroism (CD) spectroscopy.

## Experimental

### Materials and reagents

*N*-isopropylacrylamide (NIPAM, 98%), hexadecyl trimethyl ammonium bromide (CTAB, 99%), 1-vinylimidazole (VI, 99%), *N*,*N*′-methylenebisacrylamide (MBA, 99%), 3-(trimethoxysilyl) propyl methacrylate (KH-570, 97%), 2,2′-azobis (2-methylpropionamidine) dihydrochlorid (V50, 97%) and copper(ii) sulfate pentahydrate (ACS grade) were purchased from Aladdin (Shanghai, China). Guaranteed reagents of involved proteins were all purchased from Aladdin (Shanghai, China). NIPAM was recrystallized in *n*-hexane before use. All other chemicals used in the study were obtained from Sichuan Chemical Reagent Company (Chengdu, China) and used as received. Experimental deionized water was purified with Milli-Q water system with 0.4 mm filter (Millipore Corp., Bedford, USA) before use. The external magnetic field in this study was generated by a magnet with intensity of 0.5 T.

### Synthesis of Fe_3_O_4_@SiO_2_@P(NIPAM-*co*-VI)

Magnetic polymeric microspheres (Fe_3_O_4_@SiO_2_@P(NIPAM-*co*-VI)) were prepared *via* the seminal emulsion polymerization method based on the layer-by-layer process.

First, to obtain Fe_3_O_4_ MNPs with a silica-like surface that could react with various coupling agents, silica-coated Fe_3_O_4_ magnetic nanoparticles (Fe_3_O_4_@SiO_2,_ yield: 0.051 g) were first prepared according to previous studies.^28,29^ Subsequently, 0.5 g Fe_3_O_4_@SiO_2_ was used to prepared the silane coupling agent-modified Fe_3_O_4_@SiO_2_ nanoparticles (Fe_3_O_4_@SiO_2_-KH-570, yield: 0.474 g) with the vinyl groups according to our previous study^28^ and then used for seed to prepare the final composite.

Briefly, 0.5 g of Fe_3_O_4_@SiO_2_-KH-570 was placed in a 100 mL three-necked flask with 50 mL of water and dispersed by sonication. The solution was then added with 0.4802 g of NIPAM, 0.0848 g of VI, 0.0154 g of MBA, and 0.02 g of CTAB. The mixture was vigorously stirred for 30 min to obtain a mixed solution and then deoxygenated with N_2_ for another 30 min. Polymerization was carried out for 4 h in a 70 °C water bath equipped with a shaker (200 rpm) under nitrogen atmosphere. Polymerization was initiated by adding 0.05 g of V50 initiator dissolved in 10 mL of water dropwise to the aforementioned mixture. The reaction was stopped by magnetically separating the resulting magnetic particles from the reaction solution. The obtained Fe_3_O_4_@SiO_2_@P(NIPAM-*co*-VI) (yield: 0.502 g) was washed several times under sonication with deionized water and acetone, and then dried under vacuum at 45 °C until a constant weight was achieved. The final products were subsequently subjected to characterization and experimental studies.

### Synthesis of Fe_3_O_4_@SiO_2_@P(NIPAM-*co*-VI)/Cu^2+^

Exactly 50 mg of Fe_3_O_4_@SiO_2_@P(NIPAM-*co*-VI) was added to 10 mL of 0.1 M CuSO_4_ solution and mechanically stirred at room temperature for 2 h. The obtained product was separated by a magnet from the solution and washed several times with deionized water to remove unbound Cu^2+^. The resultant magnetic composites (Fe_3_O_4_@SiO_2_@P(NIPAM-*co*-VI)/Cu^2+^, yield: 0.049 g) were dried in a vacuum oven at 45 °C until a constant weight was achieved and stored for further use.

### Instruments for characterization

The morphologies of the prepared magnetic particles were studied using Scanning electron microscopy with an energy dispersive X-ray detector (SEM-EDS, GeminiSEM 300, ZEISS, Germany) and transmission electron microscopy (TEM, FEI Talos F200x, USA). Magnetic properties of the resulting samples were determined at 300 K using the MPMS-XL-7 superconducting quantum interference device (SQUID) (±7 T, Quantum Design, USA). FTIR spectra were obtained using the Spectrum Two L1600300 spectrometer (PerkinElmer, USA) in the range of 4000–400 cm^−1^. Powder X-ray diffraction (PXRD) patterns were obtained using the D8 X-ray diffractometer (Bruker, Germany) with a graphite monochromator and Cu *K*_a_ radiation (*λ* = 0.15418 nm) in the range of 10–80°. Thermal gravimetric analysis (TGA) experiments were conducted using the TG209F1 analyzer (Netzsch, Germany) at the heating rate of 10 °C min^−1^ under a N_2_ flow. The LCST of Fe_3_O_4_@SiO_2_@P(NIPAM-*co*-VI) were measured by differential scanning calorimeter (DSC, Shimadzu DSC 60) from 20 °C to 60 °C at a heating rate of 1 °C min^−1^ under nitrogen flow. The loading content of Cu^2+^ on the magnetic composites was determined by ICP-MS (NEXION 350X, PerkinElmer, USA). The surface charge of magnetic composites was determined using a Malvern Zetasizer at different pH values with 0.1 N HCl and 0.1 N NaOH electrolytes. The structure of papain was investigated using the circular dichroism spectra (CD, optical physics applications, British).

### Experiment in adsorption and desorption studies

Papain adsorption onto Fe_3_O_4_@SiO_2_@P(NIPAM-*co*-VI)/Cu^2+^ was carried out by mixing 10 mL of papain of a concentration in phosphate buffer (0.02 M) with 20 mg of magnetic composites in a 25 mL Erlenmeyer flask. The flask was incubated in an air-bath shaker with a shaking speed of 150 rpm at desired temperature (±1 °C) until the adsorption equilibrium was reached. The papain adsorption capacity of the composites, *Q* (mg papain/g particle) was calculated using eqn (1)1
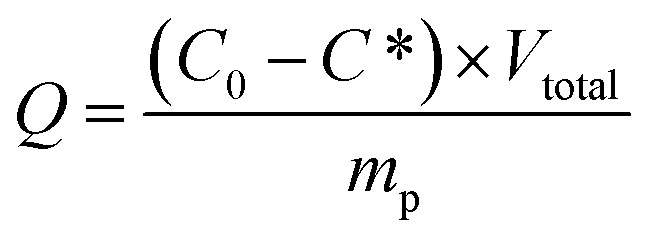
where *m*_p_ was the magnetic particles dose (g), *C*_0_ and C* (mg mL^−1^) were the papain concentration in the initial solution and in the supernatant after absorption equilibrium achieved, *V*_total_ (L) was the total volume of the sample. The protease concentration was determined by UV/vis absorption measurements at its maximum UV absorption wavelength.

After full adsorption at the optimum conditions, the papain-bonded magnetic composites were separated from the suspension by using a magnet and then washed with deionized water. Then, the papain-bonded magnetic composites were then mixed with 0.5 mL of 0.2 M imidazole solution and incubated with shaking for 30 min at 40 °C to release the papain captured by the magnetic composites. After recycling from the imidazole solution by a magnet, the collected Fe_3_O_4_@SiO_2_@P(NIPAM-*co*-VI)/Cu^2+^ were washed with phosphate buffer and sonication for 30 min by sonication for 30 min and then dried in a vacuum oven at 45 °C for reuse.

The influence of the complete adsorption and elution process on the enzymatic activity of papain was investigated by examining the activities of papain before adsorption and after elution *via* the Folin–Ciocalteu method^30^ and calculated by the standard curve of tyrosine solution obtained by an ultraviolet-visible spectrophotometer (TU-1901, Puxi Co., Ltd., Beijing, China). During the enzyme activity measurements, casein was used as a substrate. The structural changes of papain were also assessed *via* CD spectroscopy.

## Results and discussion

### Characterization of the prepared magnetic microspheres

**Fig. 1:**
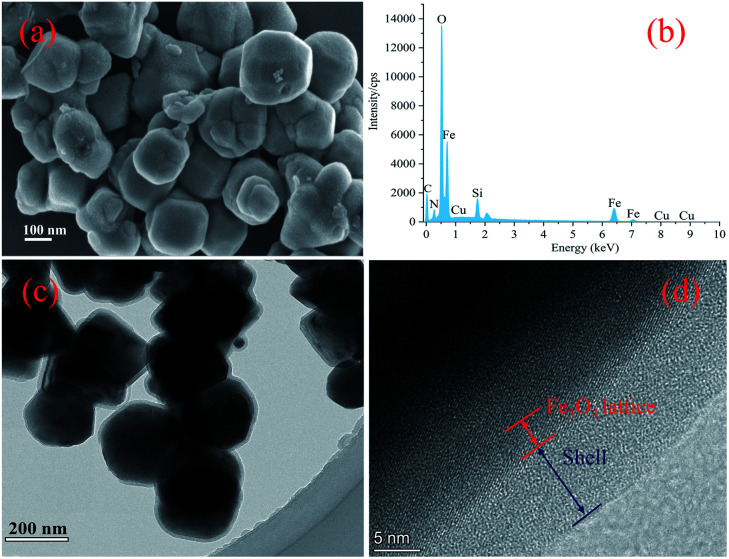
SEM-EDS results of Fe_3_O_4_@SiO_2_@P(NIPAM-*co*-VI)/Cu^2+^: (a) SEM image at 100 nm, (b) EDS spectrum (spot scanning); and TEM image of Fe_3_O_4_@SiO_2_@P(NIPAM-*co*-VI)/Cu^2+^: (c) 200 nm, (d) 5 nm.

The morphology of the Fe_3_O_4_@SiO_2_@P(NIPAM-*co*-VI)/Cu^2+^ was investigated using SEM-EDS and TEM. As shown in Fig. 1(a) and (c), the resulting magnetic composites are uniformly spherical or ellipsoidal with a stable core–shell structure. Statistical analysis indicated that the mean diameter of the microspheres is approximately 120 nm. EDS (Fig. 1(b)) revealed that Fe, Si, and Cu elements are well distributed on the surface of the magnetic composites and the mass fractions of various elements on the coating surface (wt%) were as follows: C 15%, O 26%, N 12%, Si 8%, Fe 35%, Cu 15% (the atom fractions (at%): C 8.67%, O 51.53%, N 1.34%, Si 4.06%, Fe 34.30%, Cu 9%). Moreover, compared to crystalline magnetic core, no lattice was observed on the shell (Fig. 1(d)), which is favorable for maintaining the magnetism of the nanoparticles. The PXRD pattern of magnetic composites in Fig. S1† also reveals that the layer-by-layer synthesis process did not result in a phase change of the Fe_3_O_4_ particles.

**Fig. 2:**
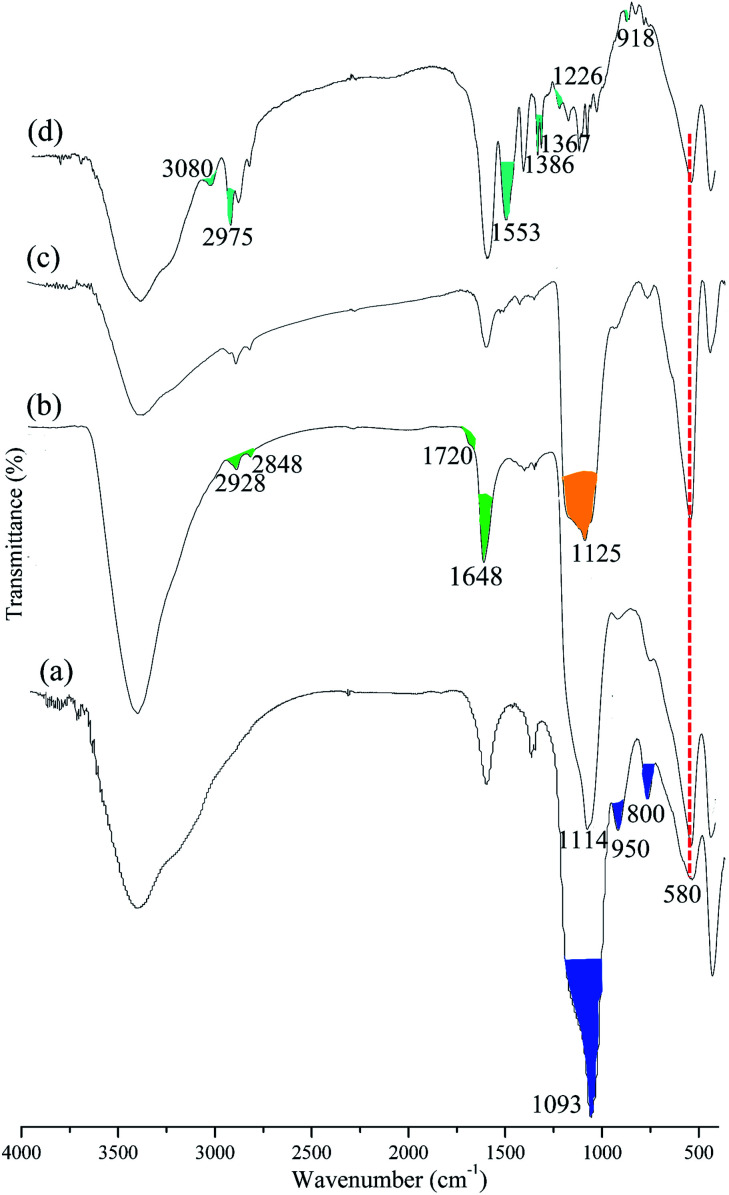
FT-IR spectra of the Fe_3_O_4_@SiO_2_ (a), Fe_3_O_4_@SiO_2_–KH-570 (b), Fe_3_O_4_@SiO_2_@P(NIPAM-*co*-VI)/Cu^2+^(c) and Fe_3_O_4_@SiO_2_@P(NIPAM-*co*-VI) (d).

The successful synthesis of the magnetic particles was confirmed by FT-IR spectroscopy. As shown in Fig. 2, the peak at approximately 580 cm^−1^ observed in all of the spectra obtained could be attributed to the characteristic vibrations of the Fe–O bond,^16^ which indicates the presence of Fe_3_O_4_ and is supported by the PXRD results (as shown in Fig. S1†). Fe_3_O_4_@SiO_2_ were prepared to achieve a silica-like surface that could react with various coupling agents. The IR spectrum of this product (Fig. 2(a)) showed the characteristic peaks of the Si–O–Si bond and Si–OH bond at 800 cm^−1^, 1093 cm^−1^ and 950 cm^−1^, which proves the formation of the silica matrix.^28,31^ After modified by silane coupling agents, absorption peaks appeared at 1720 and 1648 cm^−1^, which are related to stretch vibrations of C=O and C=C.^32^ The spectrum of Fe_3_O_4_@SiO_2_-KH-570 (Fig. 2(b)) showed absorption peaks at 2928 cm^−1^ and 2848 cm^−1^, which are due to the –CH_2_– stretch vibrations.^33^ Following further polymerization with NIPAM and VI, as seen in Fig. 2(d), two new distinctive adsorption peaks at 1386 and 1367 cm^−1^, which could be assigned to the symmetrical bending vibration of dimethyl on isopropyl, are observed. These peaks confirm the successful grafting of NIPAM units.^34^ The peaks at 918 and 1226 cm^−1^ could be attributed to the vibration of imidazolyl of the VI units. The characteristic peaks of amide I and amide II on the PNIPAM chains shifted to 1649 and 1553 cm^−1^, respectively, because of the influence of copolymer involving the C=N stretching vibration on the imidazole ring at 1552 cm^−1^.^11^ These results proved monomer VI and NIPAM was successfully coated on the surface of Fe_3_O_4_@SiO_2_. Immobilization of Cu^2+^ led to its coordination with the imidazole group,^21,22^ and its related characteristic peaks were shifted and/or overlapped (Fig. 2(c)). These observations confirm the successful synthesis of the novel magnetic composite Fe_3_O_4_@SiO_2_@P(NIPAM-*co*-VI)/Cu^2+^. The decrease in peak intensities in the PXRD patterns obtained further proves the success of surface modification.

**Fig. 3:**
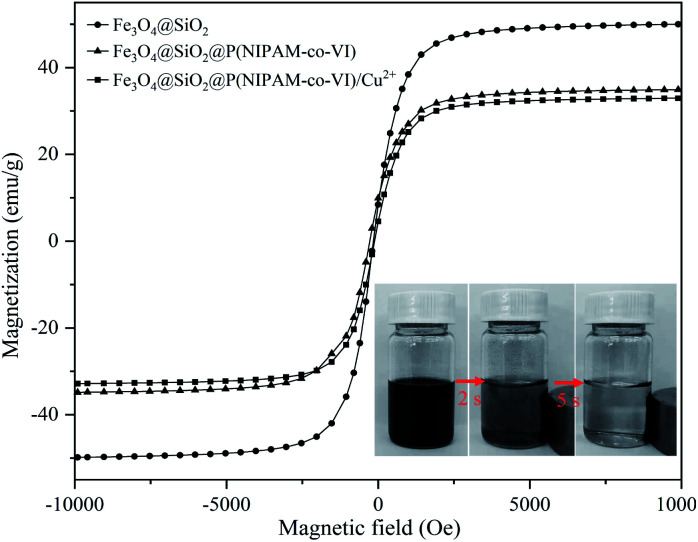
Magnetic hysteresis loops at *T* = 300 K of Fe_3_O_4_@SiO_2_, Fe_3_O_4_@SiO_2_@P(NIPAM-*co*-VI) and Fe_3_O_4_@SiO_2_@P(NIPAM-*co*-VI)/Cu^2+^, and the magnet attraction for Fe_3_O_4_@SiO_2_@P(NIPAM-*co*-VI)/Cu^2+^ with 5 s (insert picture).

Fig. 3 shows the magnetic hysteresis loops of the prepared magnetic microspheres at 300 K. All samples revealed no coersivity (*H*_C_) and no significant remanence (*M*_r_) in their magnetic hysteresis loops, which indicates super-paramagnetic behavior. The saturation magnetization values of Fe_3_O_4_@SiO_2_, Fe_3_O_4_@SiO_2_@P(NIPAM-*co*-VI) and Fe_3_O_4_@SiO_2_@P(NIPAM-*co*-VI)/Cu^2+^ were 49.98, 34.93 and 32.94 emu g^−1^, respectively. The saturation magnetization values of the materials clearly decreased after polymerization and metal-ion immobilization, which could be attributed to the presence of nonmagnetic organic substances on the surface of Fe_3_O_4_@SiO_2_. Although the saturation magnetization value of Fe_3_O_4_@SiO_2_@P(NIPAM-*co*-VI)/Cu^2+^ decreased, most of the magnetic composites were easily assembled directionally within 5 s by applying an external magnetic field with an intensity of 0.5 T (Fig. 3, inset). The magnetic property of the synthesized composite confirms its feasibility for application to magnetic separation.

**Fig. 4:**
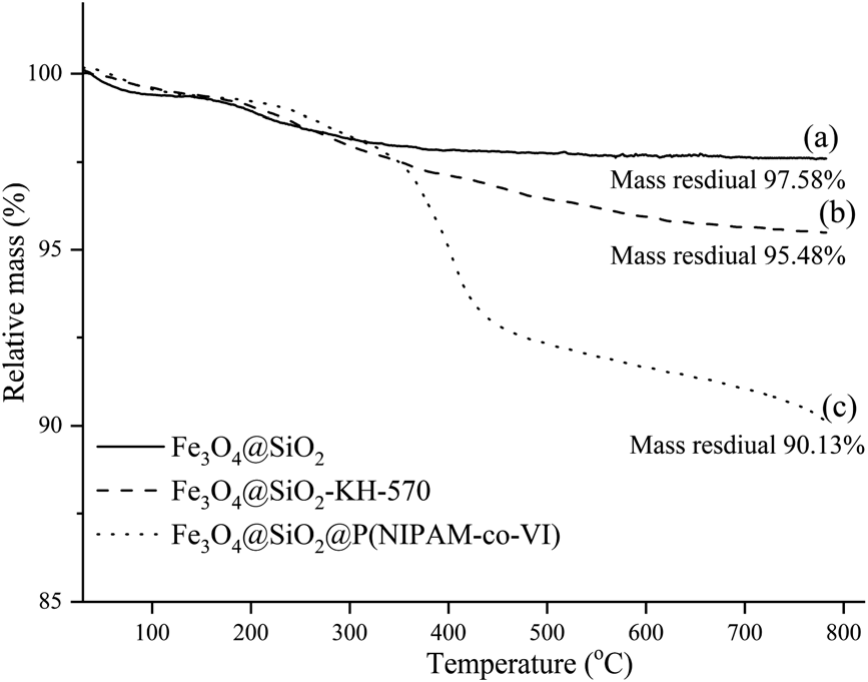
TGA curves of the Fe_3_O_4_@SiO_2_ (a), Fe_3_O_4_@SiO_2_–KH-570 (b), Fe_3_O_4_@SiO_2_@P(NIPAM-*co*-VI) (c).

The number of polymer units on the surface of the magnetic polymeric microspheres was estimated by conducting TGA, and the resulting curves are shown in Fig. 4. The TG curves obtained were similar and showed two stages of weight loss over the temperature range of 30–800 °C. The first weight loss stage was attributed to the volatilization of the residual organic solvents and the adsorbed water in the samples, while the second weight loss stages was attributed to the progressive decomposition of the grafted polymers. The remaining weight percentages obtained after TGA indicated that the grafting amount of KH-570 was 84.56 μmmol g^−1^ (2.1%, w/w) and that the amount of copolymer shell (P(NIPAM-VI)) on the composite surface is 5.35% (w/w). ICP-MS was used to estimate the loading content of Cu^2+^ on the magnetic composites, and the quantity of Cu^2+^ loaded onto the composite was calculated from the standard curve (*y* = 0.052*x*, *R*^2^ = 0.9996) to be 1.29% (w/w). According to these results and the yield of each step, the recovered fraction of magnetic beads in each step could be further calculated as 92.8%, 95.0% and 96.7%, respectively.

**Fig. 5:**
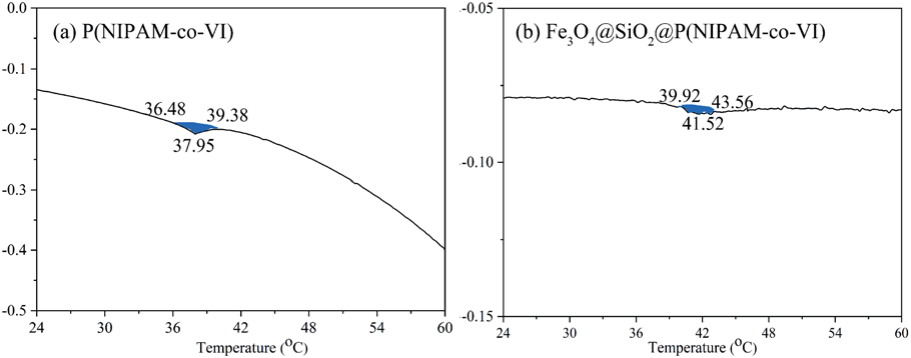
DSC curves of P(NIPAM-*co*-VI) (a) and Fe_3_O_4_@SiO_2_@P(NIPAM-*co*-VI) (b).

The thermo-responsiveness of the magnetic polymeric microspheres was evaluated by measuring the LCST of Fe_3_O_4_@SiO_2_@P(NIPAM-*co*-VI) by DSC, and the results are illustrated in Fig. 5. Compared with that of PNIPAM (LCST, ∼32 °C),^19,20^ the LCST of P(NIPAM-*co*-VI) shell shifted toward a higher temperature (37.95 °C) following the addition of the water-soluble co-monomer VI.^35^ When the Fe_3_O_4_ hybrid nanoparticle content was increased, the LCST of Fe_3_O_4_@SiO_2_@P(NIPAM-*co*-VI) increased to 41.52 °C because nanoparticle–nanoparticle interactions that makes the polymer aggregation be more difficult during the LCST.^36,37^ In addition, the volume phase transition of Fe_3_O_4_@SiO_2_@P(NIPAM-*co*-VI) occurred continuously over a narrow range of temperatures (39.92–43.56 °C), which indicates that the composite has good temperature sensitivity.^19,38^

### Optimization of conditions for papain adsorption onto the magnetic composite

#### Effect of initial concentration of papain on papain adsorption

A series of adsorption experiments was performed with different initial concentrations of papain (0.2–1.2 mg mL^−1^) (temperature 30 °C; pH 7.0). As shown in Fig. S2(a),† the adsorption capacity of the composite (mg g^−1^) could be increased by increasing the sample concentration prior to adsorption saturation; the adsorption capacity of the samples peaked when the sample concentration exceeded 1 mg mL^−1^, and the maximum adsorption amount was 158.87 mg g^−1^.

#### Effect of temperature on papain adsorption

A series of adsorption experiments was performed with different adsorption temperatures ranging from 25 °C to 55 °C (papain concentration 1 mg mL^−1^; pH 7.0). According to Fig. S2(b),† increases in the equilibrium temperature first increased and then decreased the adsorption amount of papain on the composites. The maximum adsorption amount (179.81 mg g^−1^) of papain was achieved at 35 °C. The speed of molecular motion and collision probability between papain and the magnetic composites are enhanced as the temperature increases; thus, the adsorption amount of papain also increases. DSC experiments indicated that the LCST of the magnetic polymeric microspheres is 41.52 °C, at which point the spheres undergoes hydrophilic–hydrophobic transition.^16,35^ When the temperature is lower than the LCST, the amide group of PNIPAM and water molecules produce hydrogen bonds. PNIPAM is hydrophilic and tends to swell, thereby exposing the Cu^2+^ on the composite surface and enhancing papain adsorption. However, when the temperature is higher than the LCST, hydrogen bonds between amide groups and water molecules are disrupted, and PNIPAM becomes hydrophobic. Thus, the availability of binding sites on the composites is reduced, and the adsorption amount of papain decreases.

#### Effect of pH on papain adsorption

A series of adsorption experiments was performed at different solution pH ranging from 5 to 10 (papain concentration 1 mg mL^−1^; temperature 35 °C). According to the Fig. S2(c),† the maximum adsorption amount of papain could be achieved at pH 8.0 (199.17 mg g^−1^). Increases and decreases in the solution pH beyond this value caused the adsorption amount of papain to decrease rapidly. The coordination of imidazole and Cu^2+^ dissociates under acidic conditions, resulting in a decrease in the adsorption amount of papain. Moreover, papain is a neutral protein and its pI is approximately 8.7.^39^ When the solution pH was lower than the pI, papain is positively charged, similar to the surface of the magnetic composites (Fig. S3†), and electrostatic repulsion occurs. This phenomenon in unfavorable to the adsorption process. As the solution pH increased, weak electrostatic attraction and precipitation of metal ions were observed. Thus, the adsorption amount of papain decreased.

#### Effect of ionic strength on the papain adsorption

A series of adsorption experiments was performed with different NaCl concentrations (papain concentration 1 mg mL^−1^; temperature 35 °C; pH 8.0), and the results was shown in Fig. S2(d).† The adsorption amount of papain on the magnetic composites gradually decreased from 199.17 mg g^−1^ (without NaCl) to 70.75 mg g^−1^ (1.0 M NaCl) with increase of NaCl concentration. The salting-out effect of NaCl reduces the LCST of the PNIPAM-based shell,^40^ which increases the hydrophobicity of the polymer and induces a corresponding decrease in the hydrodynamic thickness of the shell layer. Thus, the accessible surface area of the composite for papain adsorption was reduced, and a decrease in the adsorption amount of papain was observed.

### Equilibrium adsorption studies

Magnetic adsorbents play a major role in the separation process, and knowledge of the equilibrium adsorption kinetics and isotherms is fundamentally important in research on adsorption behaviors and mechanisms. Therefore, the adsorption kinetics and isotherms in the present system were investigated in detail.

#### Adsorption kinetics analysis

Adsorption is a physicochemical process that involves the mass transfer of a solute from the liquid phase to the adsorbent surface. Kinetic studies would provide important information on the mechanism of papain adsorption onto Fe_3_O_4_@SiO_2_@P(NIPAM-*co*-VI)/Cu^2+^, which is necessary to depict the adsorption rate of the adsorbate and control the residual time of the whole adsorption process. Hence, the effect of contact time on the adsorption of papain of different initial concentrations (0.4, 0.8 and 1.0 mg mL^−1^; pH 8.0) at 35 °C was investigated in this study.

As shown in Fig. S4(a),† the first 10 min of adsorption involved the initial binding of papain to the surface of the magnetic composites. The uptake of papain increased quickly thereafter. When the papain was bound to deeper active sites in the composite, the kinetic curve gradually showed a horizontal trend. The adsorption equilibrium of papain on the magnetic composites appeared within 60 min, and the maximum loading content was 199.17 mg g^−1^, which is comparably higher than that previously reported for other adsorbent materials.^41–43^

The experimental data were simulated by using the pseudo-first- and -second-order kinetic models to explore the adsorption mechanism of the magnetic composites for papain. These models are expressed as follows.

2 ln(*Q*_e_ − *Q*_t_) = ln *Q*_e_ − *k*_1_*t*/2.303

3
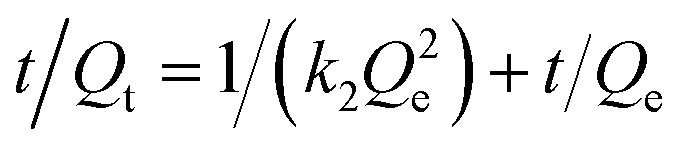
where *Q*_e_ and *Q*_t_ refer to the amount of papain adsorbed (mg g^−1^) at equilibrium and at any time *t* (min), respectively, and *k*_1_ (1/min) and *k*_2_ (g/(mg min)^−1^) is a pseudo-first-order rate constant and a pseudo-second-order rate constant, respectively.

The parameters of the two models are tabulated in Table 1. The correlation coefficients (*R*^2^) of the pseudo-second-order rate model are clearly greater than 0.9900 (see Fig. S4(b)†), and the matching degree between the adsorption amount calculated at equilibrium (*Q*_e,cal_) and the experimental data (*Q*_e,exp_) is relatively high when this model is used. This fact suggested that the rate-limiting step might be due to chemical adsorption, the high specific surface area and the absence of internal diffusion resistance.^44^ The adsorption behavior of the composites may involve valence forces arising from the sharing of electrons between papain and magnetic composites.^45^

**Table 1:** Parameters of pseudo-first order and pseudo-second order model kinetic models

*C*_0_ (mg mL^−1^)	*Q*_e,exp_ (mg g^−1^)	Pseudo-first order model			Pseudo-second order model		
		*k*_1_ (min^−1^)	*Q*_e,cal_ (mg g^−1^)	*R* ^2^	*k*_2_ (g mg^−1^ min^−1^)	*Q*_e,cal_ (mg g^−1^)	*R* ^2^
0.4	124.93	0.1128	69.03	0.9578	0.0016	126.58	0.9938
0.8	175.07	0.1257	87.57	0.9657	0.0014	178.57	0.9979
1.0	199.17	0.1131	77.70	0.9630	0.0017	200	0.9994

#### Adsorption isotherm analysis

Adsorption isotherm is critical for optimizing the use of adsorbent; these isotherms can be used to not only assess the adsorption capacity of an adsorbent but also reveal how adsorbate molecules are distributed between the liquid phase and solid phase when the adsorption process reaches equilibrium. The adsorption properties of Fe_3_O_4_@SiO_2_@P(NIPAM-*co*-VI)/Cu^2+^ for papain were evaluated by equilibrium adsorption experiments at three different temperatures (25, 30 and 35 °C) below the LCST. The results of these experiments indicated that the equilibrium adsorption capacity increases with the increasing equilibrium concentration until adsorption saturation and that the equilibrium adsorption capacity generally increases with increasing temperature (see Fig. S5(a)†).

Langmuir and Freundlich equations are two important isotherm models and widely used in absorption mechanism research. The Langmuir model assumes that there is no interaction between the adsorbate molecules and the adsorption occurs in a monolayer mode, while the Freundlich isotherm model is valid for multilayer adsorption and is derived by assuming a heterogeneous surface with interaction between adsorbed molecules with a nonuniform distribution of heat of sorption over the surface.^5,43,46^ Thus, the Equilibrium data were then analyzed using the two models, which is expressed as following, respectively.^46^4
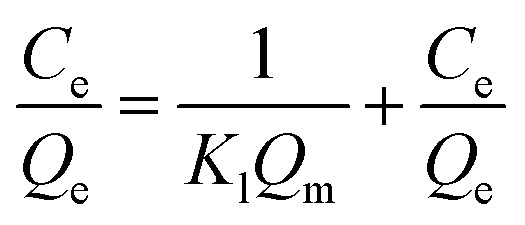

5 ln *Q*_e_ = ln *K*_f_ + ln *C*_e_/*n*

where *C*_e_ is the equilibrium concentration of papain in solution (mg mL^−1^), *Q*_e_ is the amount of papain adsorbed at equilibrium (mg g^−1^), *Q*_m_ is the maximum adsorption capacity (mg g^−1^), and *K*_l_ is the Langmuir binding constant. *K*_f_ and *n* are the Freundlich constants characteristics of the system, indicating the adsorption capacity and the adsorption intensity, respectively.

The isotherm parameters were calculated and are listed in Table 2. The high *R*^2^ values (>0.9900) obtained at all temperatures tested indicate that the equilibrium data were fitted better by the Freundlich equation than by the Langmuir equation. Thus, papain adsorption on the magnetic composites obeys the Freundlich adsorption isotherm (see Fig. S5(b)†) and occurs *via* a heterogeneous and multilayer adsorption process. The calculated Freundlich constant, 1/*n*, was lower than 1, which suggests that adsorption *via* this mechanism is a favorable process.^46,47^

**Table 2:** Parameters of Langmuir and Freundlich models

*T* (°C)	Langmuir			Freundlich		
	*Q*_m_ (mg g^−1^)	*K*_l_ (L g^−1^)	*R* ^2^	*K*_f_ (mg g^−1^)	*n*	*R* ^2^
25	161.29	0.3387	0.9875	123.92	2.6378	0.9985
30	192.31	0.2115	0.9891	162.20	2.7174	0.9980
35	270.27	0.3243	0.9753	218.42	2.2655	0.9956

#### Thermodynamics analyses

Thermodynamic parameters provide in-depth information of inherent energetic changes associated with adsorption; therefore, these parameters should be accurately evaluated. Changes in Δ*G*, Δ*H* and Δ*S* were calculated according to the ref. 48 to elucidate the adsorption process of the composites, and the results obtained are shown in Table 3. The van't Hoff plots of ln *K*_L_*versus* 1/*T* are also illustrated in Fig. S6.† The Δ*G* values obtained were negative at all temperatures evaluated, thus confirming that the adsorption process is spontaneous and thermodynamically favourable. It was also noticed that the Δ*G* increased with temperature, which shows that adsorption could be improved by increasing the system temperature. Higher temperatures appear to be able to activate more adsorption sites on the magnetic composite surface. The positive Δ*H* values obtained indicate that papain adsorption onto the magnetic composites is an endothermic process, which is supported by the increase in papain adsorption with increasing temperature. Finally, the positive Δ*S* values obtained indicate that the degrees of freedom at the solid–liquid interface increase during the adsorption process.

**Table 3:** Thermodynamic parameters for the adsorption of papain on the magnetic composites

*C*_0_ (mg mL^−1^)	Δ*H* (kJ mol^−1^)	Δ*S* (kJ mol^−1^ K)	Δ*G* (kJ mol^−1^)		
			293 K	303 K	313 K
0.2	52.19	0.223	−14.25	−15.61	−16.48
0.4	51.13	0.217	−13.42	−14.59	−15.59
0.6	48.91	0.207	−12.79	−13.95	−14.86
0.8	50.90	0.212	−12.42	−13.44	−14.55
1.0	51.52	0.213	−12.06	−13.07	−14.19

### Desorption and recyclability

**Fig. 6:**
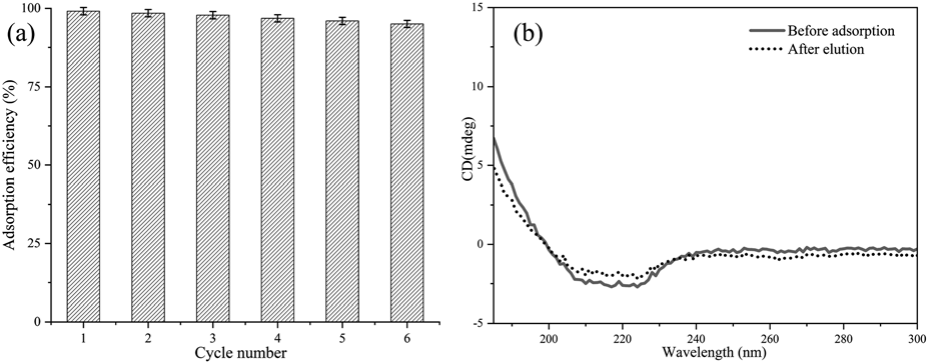
Recycling of Fe_3_O_4_@SiO_2_@P(NIPAM-*co*-VI)/Cu^2+^ in the adsorption of papain (a) and the circular dichroism spectrum of papain before the adsorption and after the elution (b).

The recyclability of magnetic adsorbents is very important to minimize costs and achieve large-scale applications. Herein, recycling experiments were performed six times (Fig. 6(a)). It is worth mentioning that the imidazole elution solution will destroy the interaction between imidazole groups and Cu^2+^, the papain was eluted and the chelated Cu^2+^ would be removed from the magnetic composites simultaneously. Thus, the magnetic composites were re-chelated to Cu^2+^ after each elution experiment and then used for the next recycling experiment. The recyclability of Fe_3_O_4_@SiO_2_@P(NIPAM-*co*-VI)/Cu^2+^ was evaluated by comparing the amount of papain collected after each experiment. The adsorption capability of papain was retained at 95% after six cycles of reuse. This finding verifies that the adsorption efficiency of the composite following further processing was well maintained, which is tremendously important to reduce costs.

The effect of the process of adsorption and elution on activity of papain was examined. The initial activity of papain was 6196 U g^−1^; after adsorption and elution, 94.6% of the activity (5862 U g^−1^) of the enzyme was retained. Moreover, the secondary structure of papain after adsorption and elution was also analyzed by CD spectroscopy, as shown in Fig. 6(b). The CD spectrum of native papain revealed a positive peak at 192 nm and two negative peaks at 208 and 222 nm. Following adsorption-elution, the corresponding peaks of the used papain remained virtually unchanged. These results reveal that the process of adsorption and elution does not significantly decrease the enzyme activity of papain.

### Thermal protection

**Fig. 7:**
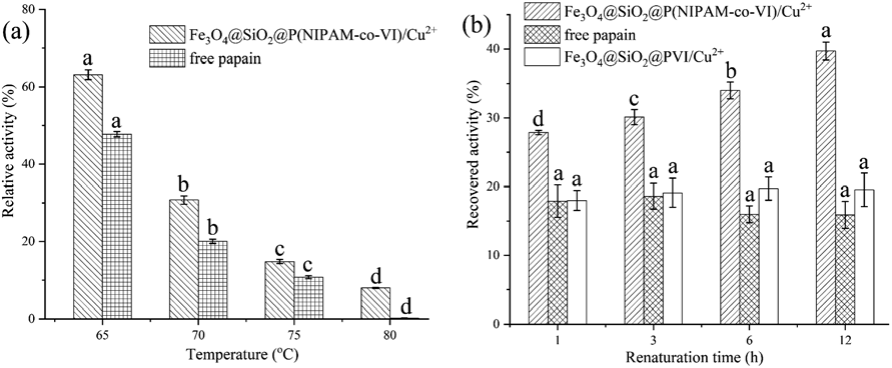
The relative activity of the papain-bonded magnetic composites and free papain after being incubated at different temperature (a) and the efficiencies of magnetic microspheres in the reactivation of papain (b). Data with different letters show significant difference (*p* <0.05).

Many existing enzymatic industrial enzymatic industrial processes are carried out at elevated temperatures; thus, heat inactivation is a critical issue in enzyme technology.^49,50^ Papain is a monomeric enzyme, and its inactivation process is divided into two steps: denaturation and deactivation.^18^ Whereas denaturation is reversible, and deactivation is irreversible. Therefore, if effective methods can be applied to prevent enzyme deactivation, the risk of loss of activity could be reduced. According to previous report,^49^ NIPAM-based materials may potentially be used to protect papain against heat inactivation. Fig. 7(a) shows the relative activity of the papain-bonded magnetic composites and free papain after being incubated at 65, 70, 75, 80 °C for 10 min. As the denaturation temperature increased, the relative activity of bound papain exceeded that of free papain; in fact, free papain nearly completely lost its activity at 80 °C. This result indicates that the magnetic composites can protect papain against heat inactivation. The main protection mechanism may be described as follows: At high temperature, enzymes are often inactivated by aggregation at hydrophobic sites which are exposed on denaturation.^51^ Thus, to prevent heat inactivation of enzymes, the key point is blocking the interactions between enzyme molecules upon denaturation.^49,52^ Under denaturation (*i.e.*, heating) conditions, PNIPAM on the surface of the magnetic composites becomes hydrophobic and forms nanoscale aggregates featuring a large specific surface area and hydrophobic surfaces. The denatured papain then coexists with PNIPAM in a confined space, which avoids the intermolecular aggregation of the solute.^49^

A series of heat denaturation/renaturation experiments was performed to investigate the thermal protection offered by the magnetic composites, and the results are shown in Fig. 7(b). Papain recovered from the magnetic composites retained nearly 40% of its activity after incubation at 70 °C for 10 min; by contrast, free papain retained only 16.3% of its activity after adsorption and elution (recovery condition: 4 °C). Magnetic microspheres without PNIPAM (Fe_3_O_4_@SiO_2_@PVI/Cu^2+^) were also prepared for comparison with Fe_3_O_4_@SiO_2_@P(NIPAM-*co*-VI)/Cu^2+^, and the results indicated that it has the same effect on papain activity as the free enzyme. These findings confirm that the prepared magnetic composites could protect the enzyme to some extent against heat inactivation. Specifically, the magnetic composites could adsorb the denatured papain at high temperatures and release it to refold at low temperatures.

## Conclusions

In the present study, a novel magnetic composite with a uniform core-shell-shell structure, Fe_3_O_4_@SiO_2_@P(NIPAM-*co*-VI)/Cu^2+^, was synthesized, characterized and evaluated for papain adsorption. The resulting magnetic composites demonstrated high adsorption capacity and could thermally protect papain. Moreover, the adsorption equilibrium results fitted the pseudo-second-order kinetic and Freundlich models well. Thermodynamic studies showed that papain adsorption onto the magnetic composites is a spontaneous and endothermic process. The results of the recycling experiments demonstrated the magnetic composites could be recycled at least six times and still retain good adsorption properties. More importantly, the complete process of adsorption and elution allowed the excellent recovery of the enzyme without loss of its activity. The results of this work present a new route to obtain novel magnetic composite-based materials for papain separation.

## Conflicts of interest

There are no conflicts to declare.

## Supplementary Material

RA-011-D1RA04128B-s001

## References

[cit1] Kirk O., Borchert T. V., Fuglsang C. C. (2002). Curr. Opin. Biotechnol..

[cit2] Choi J. M., Han S. S., Kim H. S. (2015). Biotechnol. Adv..

[cit3] Wang L., Li W., Liu Y., Zhi W., Han J., Wang Y., Ni L. (2019). Food Chem..

[cit4] Lee S. Y., Khoiroh I., Ling T. C., Show P. L. (2017). Sep. Purif. Technol..

[cit5] Han J., Wang L., Wang L., Li C., Mao Y., Wang Y. (2019). Food Chem..

[cit6] Xu J., Liu J., Che R., Liang C., Cao M., Li Y., Liu Z. (2014). Nanoscale.

[cit7] Wang B., Shao Q., Fang Y., Wang J., Xi X., Chu Q., Dong G., Wei Y. (2017). New J. Chem..

[cit8] Li M.-J., Li N., Xu G., Zhao L.-X., Chen X., Zhao Y., Zhao R.-S. (2021). Food Chem..

[cit9] Rahnama S., Shariati S., Divsar F. (2021). RSC Adv..

[cit10] Cai Z., Ye Y., Wan X., Liu J., Yang S., Xia Y., Li G., He Q. (2019). Nanomaterials.

[cit11] Paulus A. S., Heinzler R., Ooi H. W., Franzreb M. (2015). ACS Appl. Mater. Interfaces.

[cit12] Schwaminger S. P., Fraga-Garcia P., Eigenfeld M., Becker T. M., Berensmeier S. (2019). Front. Bioeng. Biotechnol..

[cit13] Zhang L., Zhu X., Jiao D., Sun Y., Sun H. (2013). Mater. Sci. Eng., C.

[cit14] Zhang Y., Yang Y., Ma W., Guo J., Lin Y., Wang C. (2013). ACS Appl. Mater. Interfaces.

[cit15] Zhen G., Falconnet D., Kuennemann E., Vörös J., Spencer N. D., Textor M., Zürcher S. (2006). Adv. Funct. Mater..

[cit16] Shamim N., Hong L., Hidajat K., Uddin M. S. (2007). Colloids Surf., B.

[cit17] Zhang M., Li Y., Yang Q., Huang L., Chen L., Ni Y., Xiao H. (2018). Carbohydr. Polym..

[cit18] Han J., Wang L., Wang Y., Cai Y., Mao Y., Ni L., Xie X. (2019). Food Bioprod. Process..

[cit19] Wang X. H., Dai Q. Q., Zhong H. Q., Liu X. X., Ren J. L. (2019). Bioresources.

[cit20] Zhou Q., Lei M., Wu Y., Zhou X., Wang H., Sun Y., Sheng X., Tong Y. (2020). Chemosphere.

[cit21] Kumar A., Khalil A. A. M., Galaev I. Y., Mattiasson B. (2003). Enzyme Microb. Technol..

[cit22] Muratalin M., Luckham P. F. (2013). J. Colloid Interface Sci..

[cit23] Barbosa-Barros L., García-Jimeno S., Estelrich J. (2014). Colloids Surf., A.

[cit24] Wong J. E., Gaharwar A. K., Muller-Schulte D., Bahadur D., Richtering W. (2008). J. Colloid Interface Sci..

[cit25] Mesquita J. P., Donnici C. L., Pereira F. V. (2010). Biomacromolecules.

[cit26] Wang J., Zhao M., Zhao Q., Jiang Y. (2007). Food Chem..

[cit27] Konno K., Hirayama C., Nakamura M., Tateishi K., Tamura Y., Hattori M., Kohno K. (2004). Plant J..

[cit28] Chen P., Yao S., Chen X., Huang Y., Song H. (2019). New J. Chem..

[cit29] Meng H., Zhang Z., Zhao F., Qiu T., Yang J. (2013). Appl. Surf. Sci..

[cit30] Oosta G. M., Mathewson N. S., Catravas G. N. (1978). Anal. Biochem..

[cit31] Zhao W., Gu J., Zhang L., Chen H., Shi J. (2005). J. Am. Chem. Soc..

[cit32] Omer M., Haider S., Park S.-Y. (2011). Polymer.

[cit33] Lv S., Song Y., Song Y., Zhao Z., Cheng C. (2014). Appl. Surf. Sci..

[cit34] Munk T., Baldursdottir S., Hietala S., Rades T., Nuopponen M., Kalliomäki K., Tenhu H., Rantanen J., Strachan C. J. (2013). Polymer.

[cit35] Pasparakis G., Tsitsilianis C. (2020). Polymer.

[cit36] Karimi A. R., Azadikhah F., Rahimi L., Ghadimi S. (2015). Colloids Surf., A.

[cit37] Rubio-Retama J., Zafeiropoulos N. E., Serafinelli C., Rojas-Reyna R., Voit B., Lopez Cabarcos E., Stamm M. (2007). Langmuir.

[cit38] Wu X. S., Hoffman A. S., Yager P. (1992). J. Polym. Sci., Part A: Polym. Chem..

[cit39] Zhu X., Zhang H. (2019). Process Biochem..

[cit40] Duracher D., Veyret R., Elaïssari A., Pichot C. (2004). Polym. Int..

[cit41] Chen T.-X., Nie H.-L., Li S.-B., Branford-White C., Su S.-N., Zhu L.-M. (2009). Colloids Surf., B.

[cit42] Szabó T., Mitea R., Leeman H., Premachandra G. S., Johnston C. T., Szekeres M., Dékány I., Schoonheydt R. A. (2008). Clays Clay Miner..

[cit43] Bian W., Yan B., Shi N., Qiu F., Lou L.-L., Qi B., Liu S. (2012). Mater. Sci. Eng., C.

[cit44] Sugawara R., Nakamura A., Murakami K. (2020). Colloids Surf., A.

[cit45] Chang Y.-C., Chen D.-H. (2005). Macromol. Biosci..

[cit46] Vimonses V., Lei S., Jin B., Chow C. W. K., Saint C. (2009). Chem. Eng. J..

[cit47] Dubey S. P., Dwivedi A. D., Sillanpää M., Gopal K. (2010). Chem. Eng. J..

[cit48] Kuo C.-Y., Wu C.-H., Wu J.-Y. (2008). J. Colloid Interface Sci..

[cit49] Tao Q., Li A., Liu X., Ma R., An Y., Shi L. (2011). Phys. Chem. Chem. Phys..

[cit50] Ó’Fágáin C. (2003). Enzyme Microb. Technol..

[cit51] Rajan R. S., Illing M. E., Bence N. F., Kopito R. R. (2001). Proc. Natl. Acad. Sci. U. S. A..

[cit52] Jiang K., Schadler L. S., Siegel R. W., Zhang X., Zhang H., Terrones M. (2004). J. Mater. Chem..

