# Supplementary material for: Fabrication of a novel core–shell–shell temperature-sensitive magnetic composite with excellent performance for papain adsorption
Source: RSC Adv. 2021 Jul 16;11(40):24843–51. doi: 10.1039/d1ra04128b (PMC9036859; doi:10.1039/d1ra04128b)
Supplement: RA-011-D1RA04128B-s001 [file RA-011-D1RA04128B-s001.pdf]

Electronic Supplementary Material (ESI) for RSC Advances.
This journal is © The Royal Society of Chemistry 2021

Supporting information for

Fabrication of a novel core-shell-shell magnetic composite with excellent performance for papain adsorption

Pengfei Chen [*a], Shun Yao[*b], Dongmei Zheng [a], Zhiyuan Xu [a], Jinling Yu [a] and Tingting Liang [a]

[a] School of Food and Bioengineering, Xihua University, Chengdu 610039, People's Republic of China.

E-mail address: pengfeimj1028@163.com

Tel/Fax: +86-28-8772-0552

[b] School of Chemical Engineering, Sichuan University, Chengdu 610065, People's Republic of China.

E-mail address: cusack@scu.edu.cn

Tel/Fax: +86-28-8540-5221

*Corresponding author

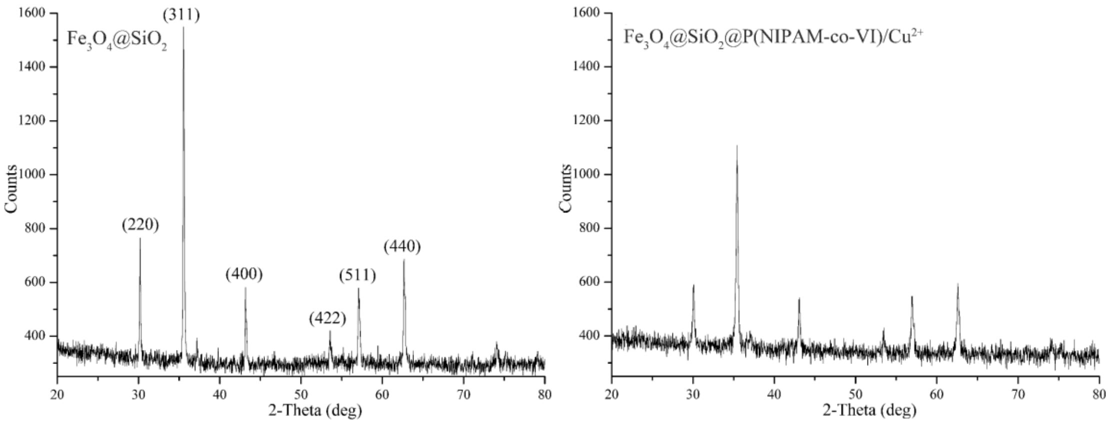

Fig. S1 PXRD spectrum of $Fe_3O_4@SiO_2$ and $Fe_3O_4@SiO_2@P(NIPAM\text{-}co\text{-}VI)/Cu^{2+}$

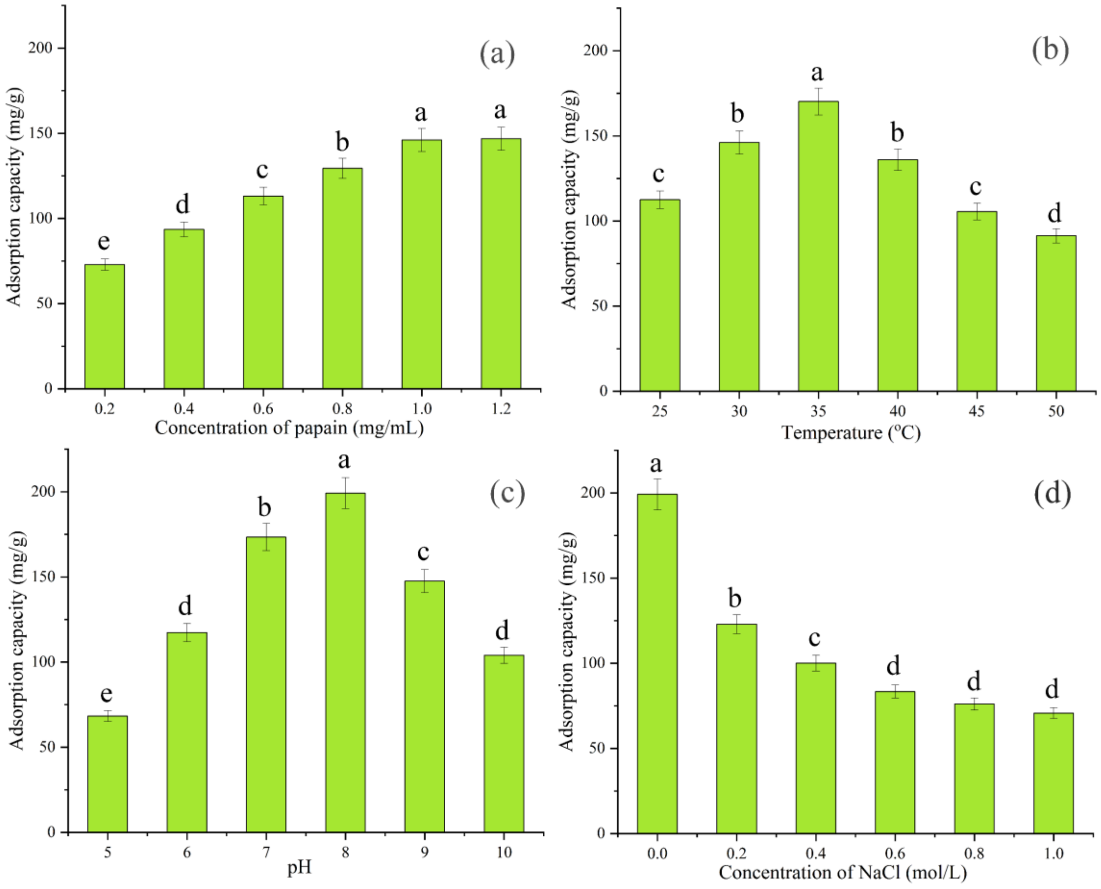

Fig. S2 Effects of the initial concentration of papain (a), temperature (b), pH (c) and concentration of NaCl (d) on the adsorption capacity of papain. Data with different letters show significant difference ($p < 0.05$).

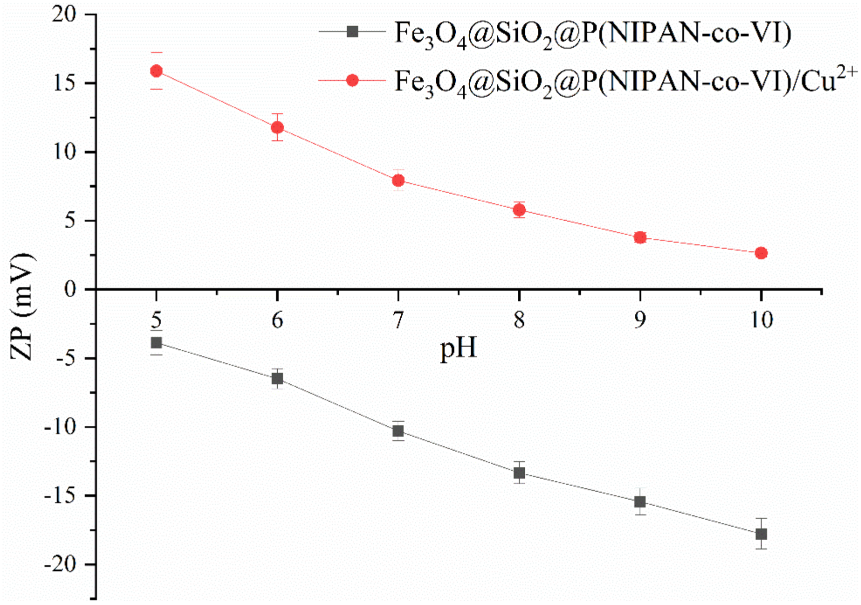

Fig. S3 Zeta-potential of $Fe_3O_4@SiO_2@P(NIPAM\text{-}co\text{-}VI)$ and $Fe_3O_4@SiO_2@P(NIPAM\text{-}co\text{-}VI)/Cu^{2+}$ at different pH values

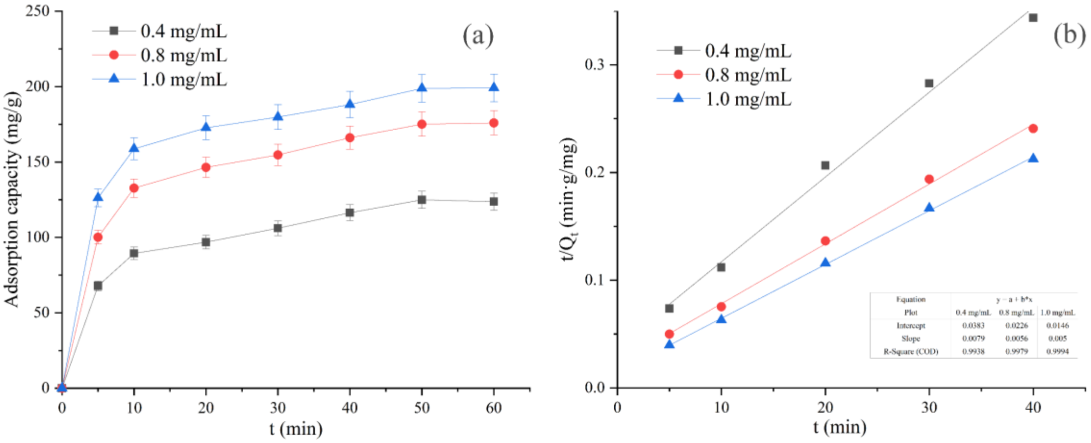

Fig. S4 The adsorption kinetics curves (a) and fitted plots of the pseudo-second order model (b) for the adsorption of papain onto the $Fe_3O_4@SiO_2@P(NIPAM\text{-}co\text{-}VI)/Cu^{2+}$

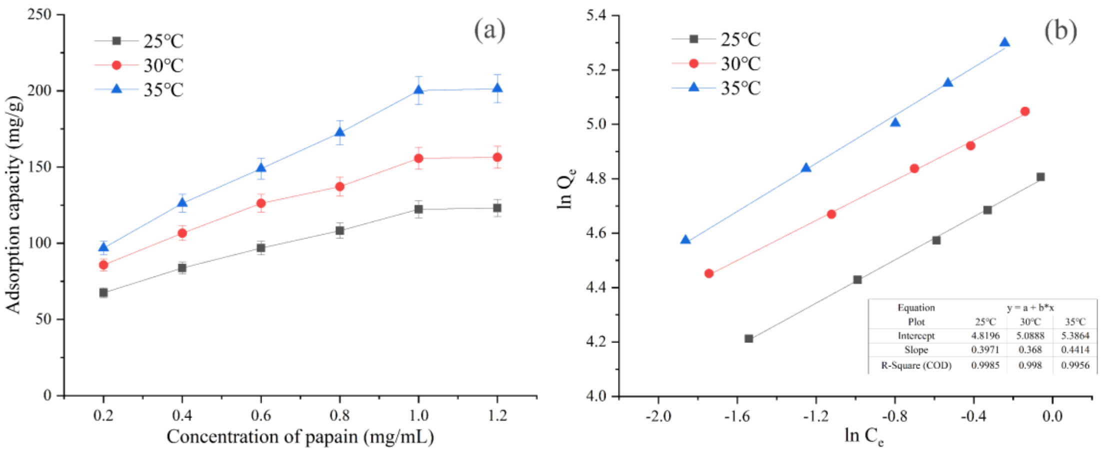

Fig. S5 The adsorption isotherms of papain at 25, 30 and 35ºC (a) and the fitted plots of Freundlich equation (b)

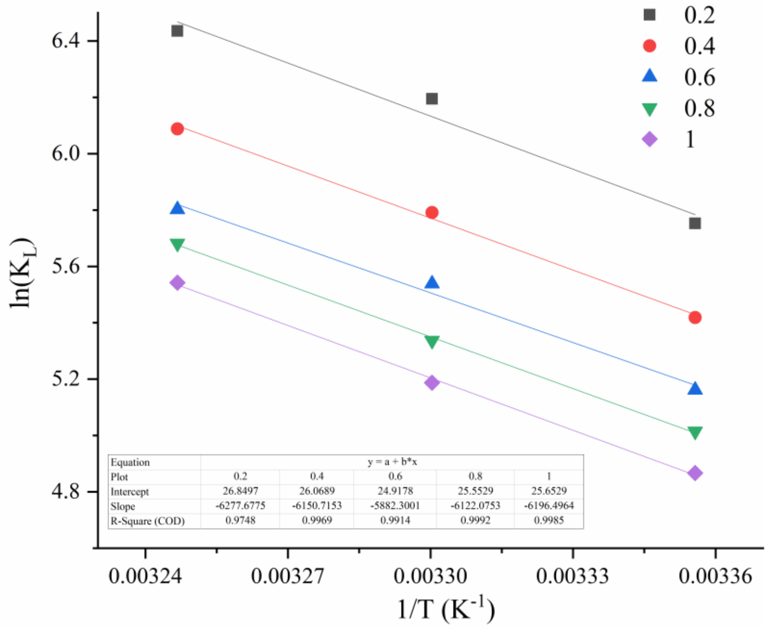

Fig. S6 The fitted plots of ln(K_L) to 1/T at different concentration of papain
